# Ostensive signals support learning from novel attention cues during infancy

**DOI:** 10.3389/fpsyg.2014.00251

**Published:** 2014-03-25

**Authors:** Rachel Wu, Kristen S. Tummeltshammer, Teodora Gliga, Natasha Z. Kirkham

**Affiliations:** ^1^Brain and Cognitive Sciences, University of RochesterRochester, NY, USA; ^2^Department of Psychological Sciences, Centre for Brain and Cognitive Development, Birkbeck, University of LondonLondon, UK

**Keywords:** infant attention, multimodal learning, eye-tracking, attentional cueing, ostensive cues

## Abstract

Social attention cues (e.g., head turning, gaze direction) highlight which events young infants should attend to in a busy environment and, recently, have been shown to shape infants' likelihood of learning about objects and events. Although studies have documented *which* social cues guide attention and learning during early infancy, few have investigated *how* infants *learn to learn* from attention cues. Ostensive signals, such as a face addressing the infant, often precede social attention cues. Therefore, it is possible that infants can use ostensive signals to learn from other novel attention cues. In this training study, 8-month-olds were cued to the location of an event by a novel non-social attention cue (i.e., flashing square) that was preceded by an ostensive signal (i.e., a face addressing the infant). At test, infants predicted the appearance of specific multimodal events cued by the flashing squares, which were previously shown to guide attention to but not inform specific predictions about the multimodal events (Wu and Kirkham, 2010). Importantly, during the generalization phase, the attention cue continued to guide learning of these events in the absence of the ostensive signal. Subsequent experiments showed that learning was less successful when the ostensive signal was absent even if an interesting but non-ostensive social stimulus preceded the same cued events.

## Introduction

By the first few months of life, infants follow social cues (i.e., head turn and gaze direction, D'Entremont, 2000; Senju and Csibra, 2008) to isolate events in a busy multimodal environment. While there is a large literature documenting when young infants begin to follow these social cues, recent work has demonstrated that social cues not only direct infants' attention, but also their subsequent learning about objects in cued locations (e.g., Yoon et al., 2008; Wu and Kirkham, 2010; Wu et al., 2011). In Wu and Kirkham (2010), 8-month-olds were presented with two identical audio-visual events simultaneously in two different locations on a computer screen. Infants' attention was oriented to one of the events using either a social cue (a face saying “Hi baby, look at this!” and turning toward the target event) or a non-social cue (a red flashing square that surrounded the target event). Both of these cues directed attention equally, as measured by equal gaze time to cued events. However, only the infants exposed to the social cue predicted the location of the cued events, suggesting that social attention cues shape the likelihood and content of learning about events during infancy.

Social cues are often preceded by ostensive signals (i.e., a smiling face making eye-contact while addressing the infant in infant-directed speech) in both natural and laboratory environments (see Csibra and Gergely, 2009). A number of studies have highlighted the importance and effectiveness of ostensive signals when directing infants' attention and learning. For example, eye contact or infant directed speech are necessary for gaze shifts to successfully orient attention in 4- and 6-month-old infants (Farroni et al., 2003; Senju and Csibra, 2008). A few recent studies suggest that ostensive signals also promote learning from gaze shifts (Wu and Kirkham, 2010; Wu et al., 2011) and pointing (Yoon et al., 2008). Ostensive signals seem to tell infants *when* to pay attention and work in conjunction with social attention cues (e.g., gaze and head direction) to tell infants *what* to learn. These signals have been suggested to enhance learning to the attended stimulus (Csibra and Gergely, 2009), although the exact underlying neural mechanisms are not known.

If ostensive signals support learning from social cues such as gaze shifts, it is possible that infants can use ostensive signals to support learning from other novel attention cues. Leekham et al. (2010) showed that by 3 years of age children were able to use a replica cue (e.g., a miniature version of a target container) to find stickers hidden underneath the actual target container only if the replica cue was presented with an ostensive signal (i.e., smiling face with eye contact). Perhaps the pairing of an ostensive signal with a novel cue is essential for infants to learn about cued events, as well as the function of the novel cue itself. Although this phenomenon has been documented during early childhood, there has yet to be a study testing whether young infants can also learn in this manner. A few infant studies, however, have shown that pairing familiar auditory social stimuli with unfamiliar auditory stimuli scaffolds learning from the latter. For example, infants are better at extracting statistical rules from sequences of non-social stimuli (e.g., tones) if they first heard those rules instantiated in social stimuli (i.e., speech; Marcus et al., 2007). Also, infants are better at word segmentation if the stimuli are presented with infant-directed rather than adult-directed speech (Thiessen et al., 2005). While these studies show that speech as a social stimulus can boost infants' learning, it is still unclear whether ostensive stimuli can help infants learn about novel visual attention cues and cued events.

We tested this hypothesis by presenting infants with a training and generalization paradigm that involved pairing a visual ostensive signal with a novel attention cue that successfully orients attention but does not produce learning about objects (Wu and Kirkham, 2010). The present eye-tracking study modified the paradigm from Wu and Kirkham (2010) with 8-month-olds. Across three experiments, infants' ability to learn from a novel attention cue (i.e., a red flashing square) following training with or without an ostensive signal was investigated. In the first experiment, infants were trained on the novel cue paired with an ostensive signal (*Ostensive Signaling*). In the second experiment, infants were given the same exposure to the novel cue in the absence of an ostensive signal (*No Signaling*). Given the large difference in stimulus presentation between including and omitting ostensive signals, Experiment 3 investigated whether including a stimulus on the screen that was social in nature but not ostensive could account for any benefits found in the Ostensive Signaling condition (*Social Non-Ostensive Signaling*).

In all three experiments, infants were familiarized with two identical dynamic multimodal objects in opposite corners of the screen and a flashing square that consistently cued the location of one of the objects. The cued familiarization trials were followed by test trials, in which infants heard the sound associated with the two objects without the appearance of the objects. Longer looking toward the previously cued location associated with the appropriate objects was taken as a measure of successful learning. Infants as young as 3 months of age succeed in this paradigm (e.g., Richardson and Kirkham, 2004; Wu and Kirkham, 2010; Kirkham et al., 2012). In the Training phase of each experiment, a different central stimulus preceded the cued events: (1) an engaging face smiling and speaking to the infant (*Ostensive Signaling*), (2) no central stimulus (*No Signaling*), or (3) two puppets speaking to each other (*Social Non-Ostensive Signaling*). The Generalization phases were identical in all three experiments, displaying only the flashing cue during the audio-visual events.

Can ostensive signals promote learning from novel cues that infants do not learn from otherwise? This study tested whether cued multimodal learning demonstrated during Test trials depended on the presence of ostensive signals during Training. Based on previous findings (e.g., Wu and Kirkham, 2010; Wu et al., 2011), we predicted that: (1) the presence of ostensive signals during Training would help infants learn to locate cued events during Test trials, (2) the presence of novel cues alone would not be sufficient for infants to show learning of the cued events, and (3) the presence of social non-ostensive signals during Training would not facilitate learning of the cued events, given their proposed lack of ability to enhance infants' learning as effectively as ostensive signals (e.g., Csibra and Gergely, 2009). Consistent with our hypothesis, we predicted that during the Generalization phase, infants trained with the ostensive signal preceding the novel cue would continue to show learning on test trials in the absence of the ostensive signal, in contrast to infants who were not exposed to this signaling.

To clarify, ostensive signals (e.g., infant-directed speech, eye-contact, smiling face) differ from social attention cues (e.g., eye gaze, head turn) because the latter directs infants' attention to a specific location. Novel attention cues in this paper refer to the flashing red square, as that was the only cue that directed attention in our study. We paired ostensive signals (that do not direct attention) with novel attention cues (that directed attention to a specific location) to investigate whether such pairing would allow infants to learn about cued events (as is the case with social attention cues, Wu and Kirkham, 2010). The social non-ostensive signal in this study refers to the muppet video used in Experiment 3.

## Experiment 1: ostensive signaling

Experiment 1 investigated the role of ostensive signals in supporting learning from a novel attention-directing cue. A dynamic face stimulus was paired with a novel flashing cue in a multimodal spatial learning paradigm. Previous research has shown that infants at this age do not learn from this attention-directing cue alone (Wu and Kirkham, 2010).

### Methods

#### Participants

Sixteen 8-month-old infants (5 girls, 11 boys, *M* = 8 months, 14 days, range: 7;24–9;12) participated in this experiment (e.g., Wu and Kirkham, 2010; Wu et al., 2011). One additional infant was excluded from analyses due to fussiness (i.e., completing only 1 out of 8 blocks). Infants were recruited via local-area advertisements and given t-shirts for participating.

#### Apparatus

Infants' looks were monitored using a Tobii 1750 eye-tracker. All dynamic stimuli were presented on the 17-inch monitor attached to the Tobii eye-tracking unit using Tobii's ClearView AVI presentation software with sounds played through stereo external speakers. The experimenter monitored whether infants were attending to the screen through an external video camera mounted on top of the Tobii screen. Infants' looks were recorded with the ClearView software. The animated object clips were created using Adobe Photoshop 7 and Macromedia Director MX 2004 (Richardson and Kirkham, 2004), and the live face clip was filmed using Macintosh iMovie (version 4.0.1). All movie clips were assembled using Final Cut Express HD 3 (Apple).

#### Stimuli and procedure

Infants sat in a car seat 60 cm from the Tobii system and eye-level to the center of the screen, while their caregivers sat behind them. A five-point infant calibration was used, and the experiment started after at least four points were correctly calibrated.

**Figure 1:**
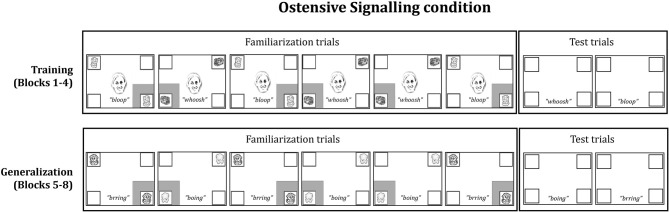
**Schematic of one block of familiarization and test trials from the *Ostensive Signaling* condition (Training and Generalization phases)**. The presentation of familiarization events was pseudo-randomized among infants (i.e., ABABBA or BABAAB), and test trial order was counterbalanced. All stimuli were in full color on a black background. The gray box around a frame represents a red flashing cue.

All infants were shown two sequences of stimuli: A *Training* phase followed by a *Generalization* phase. Within each phase, infants saw four blocks of stimuli, each of which consisted of six familiarization trials and two test trials (see Figure 1 for a schematic and examples of the stimuli).

***Training phase*.** The familiarization trials in the Training phase began with a 4-s video clip of an ostensive signal, which was presented in the center of the screen. The video subtended 2.86 × 4.29°, where approximately half of the scene comprised of the face. We used the initial ostensive stimulus from Wu and Kirkham (2010) and Wu et al. (2011) as the ostensive signal in this study: A female face looked at the infant, said, “Hi baby, look at this!,” and froze as a still image with a smile directed at the infant. Then, a pair of identical audio-visual objects appeared inside white square frames, which were located in diagonally opposing corners of the screen (e.g., lower right and upper left) and subtended 2.39 × 2.39°. Following the trial setup of Wu and Kirkham (2010), a red flashing square (i.e., the novel attention cue) appeared simultaneously with the audio-visual events surrounding the lower object. The face did not offer any directional information because it only spoke and looked out at the infant without turning or shifting gaze. The two identical multimodal objects and the frozen face remained on the screen until the end of the trial 5 s later. A stationary kaleidoscopic attention getter with ringing sounds played between each trial to re-engage the infant with the screen. Across the training phase, there were two different pairs of multimodal objects (e.g., two cats making *bloop* sounds, and two buses making *whoosh* sounds) with each pair appearing on three out of six familiarization trials per block. One pair appeared in the bottom left and top right frames on half of the trials, while the other pair appeared in the bottom right and top left frames on the other half of the trials.

After six familiarization trials, two test trials were presented. Test trials consisted of a blank screen containing only the empty white frames. During each test trial, the sound associated with a particular pair of objects played for 5 s (e.g., the *bloop* sound associated with the two cats), while the four white frames remained empty in the corners of the screen. After the test trials, the next block began. Infants saw four blocks of trials, each consisting of a succession of six familiarization trials and two test trials. The same two pairs of audio-visual events were shown for all four blocks within the Training phase. Presentation of the pairs was randomized within subjects, and pair locations were counterbalanced between subjects.

***Generalization phase*.** The Generalization phase immediately followed the Training phase. No central cues were presented during familiarization trials in the Generalization phase, so they were 4 s shorter than the familiarization trials in the Training phase. The familiarization trials in the Generalization phase displayed two new audio-visual pairs (e.g., two ducks making *brring* sounds and two dogs making *boing* sounds), which were presented with a single red flashing square surrounding one of the two events on a given trial. The audio-visual events were counterbalanced between participants, such that half the infants saw the pairs of audio-visual animations during Training that the other half saw during Generalization. Mirroring the Training phase sequence, the Generalization phase each consisted of six familiarization trials followed by two test trials repeated over four blocks.

#### Data reduction and analysis

**Figure 2:**
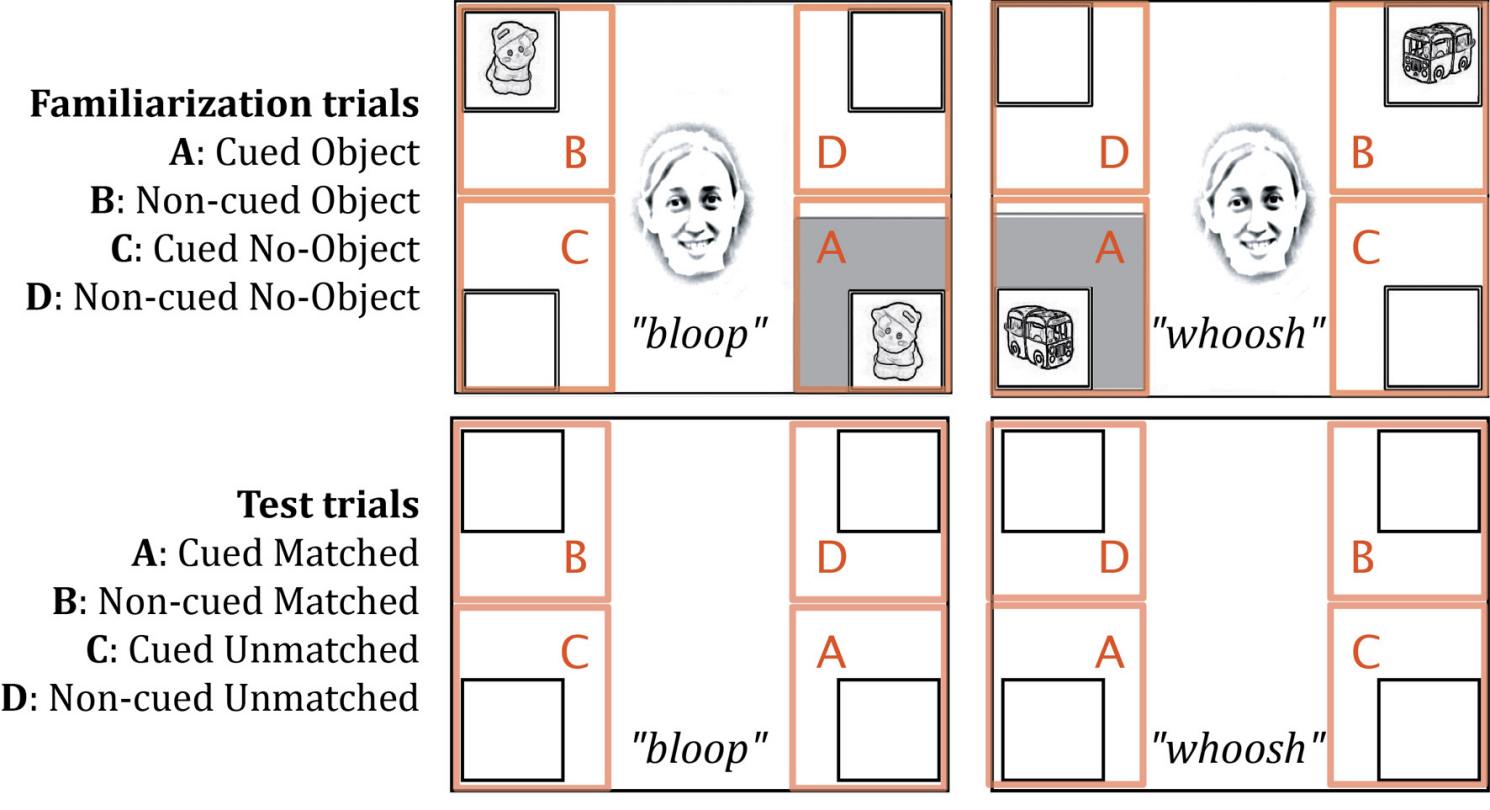
**Areas of interest (AOIs) delineated for familiarization and test trials**. The four AOIs were identical in area.

Data were acquired and analyzed using Tobii's ClearView software. Within each trial, two of four framed locations contained objects that were paired with a particular sound (a bottom corner and the opposite diagonal corner; see Figure 2). Across trials, two different locations were cued (bottom right or bottom left corner of the screen, depending on which pair of animations was present). Thus, four areas of interest (AOIs, see Figure 2) were manually delimited for all trials around the four corner frames. We measured the accumulated looking time within each of these locations for the 5 s during which audio-visual events were visible (or in the case of test trials, the corresponding 5 s during which accompanying sounds were played). The standard temporal filter of 100 ms and spatial filter of 30 pixels were used to define fixations. For each AOI, we reported the proportional looking time, which was calculated in each trial for every infant by dividing the total looking time in that AOI by the total looking time in all four AOIs.

Given our prediction of ostensive signals supporting learning from novel attention cues, we analyzed the two phases (Training and Generalization) separately. This allowed us to analyse the effect of training (differing only in the central stimulus prior to the learning events) on generalization across experiments. Within each phase we investigated looking behavior during the familiarization trials and the test trials separately. While the analysis of the familiarization trials allowed us to describe the distribution of attention in response to the presence of ostensive signals and flashing cues, the analyses of the test trials contribute the crucial evidence for cued learning. Infants had to integrate two sources of information during test trials: the location of multimodal objects, and the location of cued events. Accordingly, analyses examined the effects of Object (increased looking to the diagonally opposite locations that contained identical objects) and Cue (increased looking to the locations that were surrounded by red flashing square cues). The following outcomes were possible: (1) a significant Cue × Object interaction, indicating that infants learned about cued object-sound pairings, (2) a main effect of Object, such that infants looked more at both cued and non-cued locations of the objects paired with the corresponding sound, or (3) a main effect of Cue, showing that infants looked equally at both cued locations, independent of where the objects had appeared. The absence of any effects would indicate that infants distributed their looking equally to all four locations and did not learn from this paradigm. A significant Cue × Object interaction was followed up by a planned *post-hoc t*-test that compared looking to the Cued and Non-cued object locations.

### Results

#### Ostensive Signaling condition: training phase

**Figure 3:**
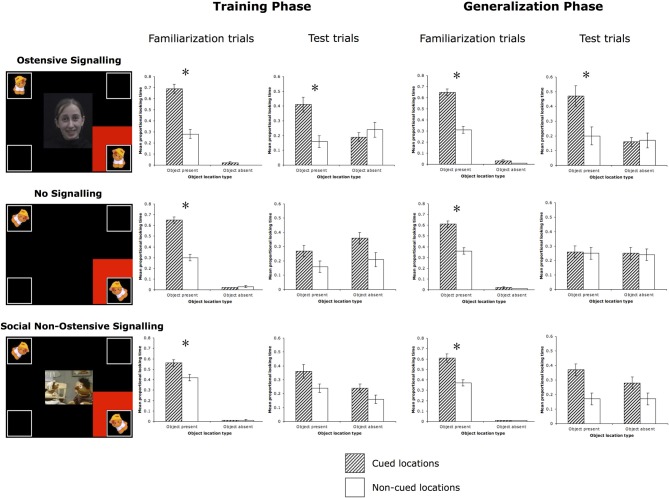
**Familiarization and test trials for all three conditions**. All stimuli were in full color on a black background. The bar graphs display the results from the familiarization and test trials. ^*^p < 0.03.

***Familiarization trials*.** A 2-Way (Cued location × Object location) within-subjects ANOVA revealed main effects of Cue [*F*_(1, 15)_ = 27.98, *p* < 0.001, partial η^2^ = 0.65] and Object [*F*_(1, 15)_ = 1578.23, *p* < 0.001, partial η^2^ = 0.99], and a significant interaction between the two [*F*_(1, 15)_ = 28.44, *p* < 0.001, partial η^2^ = 0.66]. As expected, infants followed the cue to the targeted object and spent more time looking at it than at the identical object in the diagonally opposite location, planned *post-hoc*: *t*_(15)_ = 5.37, *p* < 0.001, Cohen's *d* = 2.56 (Table 1, Figure 3).

**Table 1:** **Mean proportional looking times during familiarization and test trials to four areas of interest (AOIs) in the Ostensive Signaling, No Signaling, and Social Non-Ostensive Signaling conditions**.

**Locations (AOIs)**	**Condition**											
	**Ostensive Signaling condition**				**No Signaling condition**				**Social Non-Ostensive Signaling condition**			
	**Training**		**Generalization**		**Training**		**Generalization**		**Training**		**Generalization**	
	***M***	***SE***	***M***	***SE***	***M***	***SE***	***M***	***SE***	***M***	***SE***	***M***	***SE***
**FAMILIARIZATION TRIALS**												
Cued object	0.69	0.04	0.65	0.03	0.65	0.03	0.61	0.03	0.56	0.03	0.61	0.04
Non-cued object	0.28	0.04	0.31	0.03	0.30	0.03	0.36	0.03	0.42	0.03	0.37	0.03
Cued no-object	0.02	0.01	0.03	0.01	0.02	0.00	0.02	0.01	0.01	0.00	0.01	0.00
Non-cued no object	0.00	0.00	0.01	0.00	0.03	0.01	0.01	0.00	0.01	0.01	0.01	0.00
**TEST TRIALS**												
Cued matched	0.41	0.05	0.47	0.07	0.27	0.04	0.26	0.04	0.36	0.05	0.37	0.04
Non-cued matched	0.16	0.04	0.20	0.06	0.16	0.04	0.25	0.04	0.24	0.03	0.17	0.04
Cued unmatched	0.19	0.03	0.16	0.03	0.36	0.04	0.25	0.04	0.24	0.03	0.28	0.04
Non-cued unmatched	0.24	0.05	0.17	0.05	0.21	0.05	0.24	0.04	0.16	0.03	0.17	0.04

***Test trials*.** A 2 (Cued location) × 2 (Object location) ANOVA yielded a trend toward a significant main effect of Cue [*F*_(1, 15)_ = 4.19, *p* = 0.06, partial η^2^ = 0.22], no main effect of Object [*F*_(1, 15)_ = 2.18, *p* = 0.16, partial η^2^ = 0.13], and a significant Cue × Object interaction [*F*_(1, 15)_ = 9.11, *p* = 0.01, partial η^2^ = 0.38]. Based on the significant interaction, planned *post-hoc* comparisons [*t*_(15)_ = 3.37, *p* = 0.004, Cohen's *d* = 1.39] revealed that infants looked longer to the correct object location that had previously been cued during test trials.

#### Ostensive Signaling condition: generalization phase

***Familiarization trials*.** A 2 (Cued location) × 2 (Object location) ANOVA yielded main effects of Cue [*F*_(1, 15)_ = 26.28, *p* < 0.001, partial η^2^ = 0.64], and Object [*F*_(1, 15)_ = 2629.98, *p* < 0.001, partial η^2^ = 0.99], and a significant interaction between the two [*F*_(1, 15)_ = 24.44, *p* < 0.001, partial η^2^ = 0.62]. Again, as expected, infants followed the cues to the targeted object, looking longer at it than at the identical object in the diagonally opposite location, planned *post-hoc* Cued Object vs. Non-cued Object: *t*_(15)_ = 5.09, *p* < 0.001, Cohen's *d* = 2.13.

***Test trials*.** A 2 (Cued location) × 2 (Object location) ANOVA yielded a trend toward a significant main effect of Cue [*F*_(1, 15)_ = 3.39, *p* = 0.086, partial η^2^ = 0.18], a significant main effect of Object [*F*_(1, 15)_ = 9.64, *p* = 0.007, partial η^2^ = 0.39], and a significant Cue × Object interaction [*F*_(1, 15)_ = 6.09, *p* = 0.03, partial η^2^ = 0.29]. Infants continued to look longer at the correct object location that had previously been cued during familiarization compared to the non-cued correct object, planned *post-hoc*: *t*_(15)_ = 2.43, *p* = 0.03, Cohen's *d* = 1.04.

### Discussion

Infants' attention was successfully directed to the cued audio-visual event during the familiarization trials in both the Training and the Generalization phases. During test trials in the Training phase, when infants were presented with four blank white frames and the sound from one of the audio-visual pairs, infants looked to the appropriate cued locations. They associated the correct sound with the cued locations where the corresponding objects had previously appeared. This result extends previous findings showing the same type of learning with ostensive signals paired with social attention cues (head turn and gaze shift; Wu and Kirkham, 2010). Infants' performance during test trials in the Generalization phase was also consistent with our hypothesis. Infants looked to the cued correct location that had been previously associated with the presented sound. In Wu and Kirkham (2010) infants of a similar age did not learn the multimodal pairing when presented with only the red flashing square as a cue. Therefore, we suggest that the addition of the Training phase that paired the ostensive signal with the flashing cue could have supported learning from the flashing cue during both the Training and Generalization phases. There is, however, an alternative hypothesis: Perhaps just extended exposure to the red flashing square cue could have supported learning at least by the Generalization phase, and the preceding ostensive signal was not necessary for specific multimodal learning. The following *No Signaling* experiment was undertaken to investigate this alternative hypothesis.

## Experiment 2: no signaling

In Experiment 2, a new group of infants were presented with identical stimuli as in Experiment 1, with one critical difference—the absence of ostensive signals during the Training Phase. Thus, in this experiment, infants saw two similar Training and Generalization phases. This experiment tested the alternative hypothesis that extended exposure to the novel cue is sufficient for infants to learn about the audio-visual events. It is possible that although the ostensive signal can support learning from the flashing squares, mere extended exposure to the novel attention cue could also support this learning.

### Methods

#### Participants

A separate group of sixteen 8-month-old infants (10 girls, 6 boys, *M* = 8 months, 21 days, range: 7;29–9;21) composed the final sample in this condition. One additional infant was excluded from the final analyses due to fussiness (i.e., completing only 1 out of 8 blocks).

#### Apparatus, stimuli, and procedure

All aspects of this experiment were nearly identical to Experiment 1; infants in the *No Signaling* experiment were shown the same flashing red squares and audio-visual events as infants in the *Ostensive Signaling* experiment. There was, however, no centrally-presented video of a face appearing before any of the events, making the familiarization trials during Training 4 s shorter than those in the *Ostensive Signaling* experiment. In other words, the Training phase and the Generalization phase were identical in this experiment, except for the differing audio-visual events.

### Results

As in Experiment 1, there were four possible outcomes: (1) learning about the cued object (Cue × Object interaction), (2) learning about the audio-visual objects regardless of cued locations (main effect of Object), (3) learning only about cued locations regardless of multimodal information (a main effect of Cue), or (4) no learning (looking equally to all four locations). Given that the Training phase in this study had the same procedure as previous work (Wu and Kirkham, 2010), we predicated infants would look only to cued locations during test trials (main effect of Cue).

#### No Signaling condition: training phase

***Familiarization trials*.** A 2-Way (Cued location × Object location) within-subjects ANOVA revealed main effects of Cue [*F*_(1, 15)_ = 28.987, *p* < 0.001, partial η^2^ = 0.66] and Object [*F*_(1, 15)_ = 3602.87, *p* < 0.001, partial η^2^ = 0.996], and a significant Cue × Object interaction: *F*_(1, 15)_ = 47.40, *p* < 0.001, partial η^2^ = 0.76. Similar to Experiment 1, during familiarization trials with the red flashing square cue, infants followed the cues to the targeted event and spent more time looking at it than at the identical event in the diagonally opposite location, planned *post-hoc*: *t*_(15)_ = 6.14, *p* < 0.001, Cohen's *d* = 2.92 (Table 1, Figure 3).

***Test trials*.** A 2 (Cued location) × 2 (Object location) ANOVA yielded no significant effects or interaction. There was a trend toward a significant main effect of Cue, *F*_(1, 15)_ = 3.41, *p* = 0.09, partial η^2^ = 0.19, with infants looking longer at the cued locations (the two bottom corners) vs. the non-cued locations (the two top corners), but they did not look to appropriate object locations based on which sound was playing.

#### No Signaling condition: generalization phase

***Familiarization trials*.** A 2 (Cued location) × 2 (Object location) ANOVA yielded main effects of Cue [*F*_(1, 15)_ = 14.51, *p* = 0.002, partial η^2^ = 0.49], and Object [*F*_(1, 15)_ = 6315.74, *p* < 0.001, partial η^2^ = 0.998], and a significant Cue × Object interaction [*F*_(1, 15)_ = 14.35, *p* = 0.002, partial η^2^ = 0.49]. Again, as expected, infants followed the cues to the targeted object, looking longer at it than at the identical object in the diagonally opposite location, planned *post-hoc* Cued Object vs. Non-cued Object: *t*_(15)_ = 3.81, *p* = 0.002, Cohen's *d* = 2.08.

***Generalization phase*.** A 2 (Cued location) × 2 (Object location) ANOVA revealed no significant effects or interactions, all *F* < 0.2.

### Discussion

Although infants' attention was successfully directed to the cued audio-visual event during the familiarization trials, in both the Training and the Generalization phases, the test trials did not reveal any multimodal learning. A trend toward looking at the bottom corners appeared during the Training phase (i.e., the cued locations, similar to Wu and Kirkham, 2010), but no specific multimodal learning was seen (i.e., looking at the correctly cued corner when a specific sound was played). The trend did not appear during the Generalization phase. This suggests that mere extended exposure to the novel cue was not enough to support appropriate learning of the audio-visual events. Rather, it seems that the ostensive signal in Experiment 1 initially paired with the flashing cues may be necessary to support this learning.

The training phases of Experiments 1 and 2 differed not only in the presence or absence of ostensive signals, but most notably in the presence or absence of any central stimulus. Perhaps including a central stimulus on the screen that was social in nature but not ostensive could account for any benefits found in the *Ostensive Signaling* condition compared to the *No Signaling* condition. The aim of Experiment 3 was to address this issue.

## Experiment 3: social Non-Ostensive Signaling

Compared to Experiment 1, this experiment presented a different central stimulus immediately before the audio-visual events and the red flashing squares: Instead of a female face providing ostensive signals, a new group of infants saw a video of two Sesame Street puppets interacting with each other. This experiment tested the alternative hypothesis that any social non-ostensive stimulus in the center prior to the flashing cues would facilitate learning of the cued audio-visual events. It could be that ostensive signals (direct eye gaze, smiling face, infant-directed speech) are not necessary for boosting cued learning, and that any social non-ostensive stimulus would provide similar results.

### Methods

#### Participants

A separate group of seventeen 8-month-old infants (7 girls, 10 boys, *M* = 8 months, 18 days, range: 7;20–9;23) composed of the final sample in this experiment. One additional infant was excluded from the final analyses due to experimenter error (i.e., playing the stimuli in the wrong sequence).

#### Apparatus, stimuli, and procedure

All aspects of this experiment were identical to Experiment 1 with the exception of the following: Instead of a face as the central stimulus, the familiarization trials during Training showed a 4-s clip from *Sesame Street* in which Ernie says to another muppet, “*I'm going to teach you to say something very important*.” At the end of the 4 s, the video stopped to a still frame and the audio-visual events then appeared with the red flashing square, highlighting one of the events. The central stimulus was chosen because it contained speech (social stimulus), but was not directed at the infant participant (and therefore was not an ostensive signal) and provided no additional information to the infants about where to look. We also chose the stimulus because infants found it very engaging in previous studies (e.g., Wu and Kirkham, 2010; Wu et al., 2011) as a standard Tobii infant calibration video. The video clip subtended 5.25 × 4.29°, where approximately half of the scene comprised of the muppets. The length of all trials in this condition matched those in Experiment 1.

### Results

The data were analyzed in the same manner as in Experiment 1. As in Experiments 1 and 2, there were four possible outcomes: (1) learning about the cued object, (2) learning about the audio-visual objects regardless of cued locations, (3) learning only about cued locations regardless of multimodal information, or (4) no learning. If this social non-ostensive stimulus was just as effective as the ostensive stimulus in Experiment 1, infants would show a Cue × Object interaction during test trials in both the Training and Generalization phases.

#### Social Non-Ostensive Signaling condition: training phase

***Familiarization trials*.** A 2-Way (Cued location × Object location) within-subjects ANOVA revealed main effects of Cue [*F*_(1, 16)_ = 5.73, *p* = 0.03, partial η^2^ = 0.26] and Object [*F*_(1, 16)_ = 8051.08, *p* < 0.001, partial η^2^ = 0.998], and a significant interaction between the two [*F*_(1, 16)_ = 6.83, *p* = 0.02, partial η^2^ = 0.30]. Infants performed similarly to the previous two experiments, looking longer to the cued object than to the non-cued object (in the diagonally opposite corner), planned *post-hoc*: *t*_(16)_ = 2.51, *p* = 0.02, Cohen's *d* = 1.13 (Table 1, Figure 3).

***Test trials*.** A 2 (Cued location) × 2 (Object location) ANOVA revealed a main effect of Object [*F*_(1, 16)_ = 5.15, *p* = 0.04, partial η^2^ = 0.24] and a marginal main effect of Cue [*F*_(1, 16)_ = 3.76, *p* = 0.07, partial η^2^ = 0.19]. Infants looked longer to cued compared to non-cued locations as well as object compared to empty locations, but the interaction between Cue and Object was not significant [*F*_(1, 16)_ = 0.65, *p* = 0.43, partial η^2^ = 0.04]. This suggests that infants looked more to the two correct object corners, as well as the cued corners, but did not look more to the specific correct cued object corner.

#### Social non-ostensive signaling condition: generalization phase

***Familiarization trials*.** A 2-Way (Cued location × Object location) within-subjects ANOVA revealed main effects of Cue [*F*_(1, 16)_ = 10.92, *p* = 0.004, partial η^2^ = 0.41] and Object [*F*_(1, 16)_ = 11829.53, *p* < 0.001, partial η^2^ = 0.999], and a significant interaction between the two [*F*_(1, 16)_ = 12.69, *p* = 0.003, partial η^2^ = 0.44], which is again similar to the previous two experiments, suggesting that attention was being directed successfully to the cued object location, planned *post-hoc* Cued Object vs. Non-cued Object: *t*_(16)_ = 3.44, *p* = 0.003, Cohen's *d* = 1.66.

***Test trials*.** A 2 (Cued location) × 2 (Object location) ANOVA revealed only a significant effect of Cue [*F*_(1, 16)_ = 15.22, *p* = 0.001, partial η^2^ = 0.49], indicating that infants looked longer at previously cued locations. However, there was no significant main effect of Object [*F*_(1, 16)_ = 0.62, *p* = 0.44, partial η^2^ = 0.04], nor an interaction [*F*_(1, 16)_ = 2.78, *p* = 0.12, partial η^2^ = 0.15].

#### Cross-experiment comparisons

Compared to infants in the Ostensive Signaling condition, infants in the Social Non-Ostensive Signaling condition looked marginally less to the central stimulus, *F*_(1, 31)_ = 3.89, *p* = 0.057, partial η^2^ = 0.21, *M*_NoOst_ = 85.88 s, *SE*_NoOst_ = 6.19, *M*_Ost_ = 104.24 s, *SE*_Ost_ = 6.98, but had similar looking times throughout the entire experiment, *F*_(1, 31)_ < 0.12, *M*_NoOst_ = 182.49 s, *SE*_NoOst_ = 18.21, *M*_Ost_ = 175.09 s, *SE*_Ost_ = 11.14. Furthermore, infants in all three experiments looked equally long at the cued object during familiarization, *F* < 0.5, *M*_Ost_ = 49.55 s, *SE*_Ost_ = 5.17, *M*_NoSig_ = 52.82 s, *SE*_NoSig_ = 4.71, *M*_NoOst_ = 46.35 s, *SE*_NoOst_ = 4.31. Infants did differ across experiments in proportional looking times to the cued object during test trials of the Training phase, as shown in a significant Cued location × Object location × Experiment interaction, *F*_(2, 45)_ = 6.24, *p* = 0.004, partial η^2^ = 0.22, and similarly during test trials of the Generalization phase [*F*_(2, 44)_ = 2.78, *p* = 0.073, partial η^2^ = 0.11].

### Discussion

Although infants in the *Social Non-Ostensive Signaling* experiment successfully followed the novel cues during familiarization trials in both the Training and the Generalization phases, the test trials revealed only non-specific learning. In the Training phase test trials, infants looked significantly longer to the two locations where the objects had appeared when their corresponding sound was played (i.e., a bottom corner and its diagonally opposite corner), compared to the two locations that were not paired with the sound. There was also a trend toward looking to the cued corners more than the non-cued corners. However, the absence of a Cued location × Object interaction suggests that infants did not look consistently to the cued corner that had contained an object. Rather their looks were more distributed among the three “Object” and “Cued” locations. In the Generalization phase test trials, infants looked to the two cued corners, but did not choose the location where the corresponding object had previously appeared, similar to infants in the Training Phase in the No Signaling condition. This indicates that training with a social non-ostensive signal preceding a novel flashing cue was not as effective in focusing learning on a particular event. That is, attention remained distributed among multiple visual events rather than directed toward one particular target, even though infants attended to that target during the familiarization trials of both the training and generalization phases. The social non-ostensive signal appears to have initially directed infants to learn about the multimodal objects, as well as the cued locations, without linking the two pieces of information. Further, when the non-ostensive signal disappeared in the generalization phase, the infants only learned about the cued locations, rather than the multimodal events.

In summary, ostensive signals seemed to help infants learn about cued multimodal events during training with the signal, as well as during generalization without the signal. Without this initial signal (*No Signaling*, Experiment 2), or even when substituted by a social non-ostensive signal (*Social Non-Ostensive Signaling*, Experiment 3), infants did not demonstrate location-specific audio-visual learning.

## General discussion

The present study provides preliminary evidence for ostensive signals helping infants learn from novel attention-directing cues (i.e., flashing squares). The measure of learning used was the proportion of infants' looking times to the previously cued location of an audio-visual event when only the associated sound (not visual stimulus) was presented. When the novel attention-directing cue was paired with an ostensive signal, 8-month-olds predicted the events would appear in the appropriate cued locations, even though the face had not offered any directional information (i.e., the head or the gaze never turned toward the cued location). After initial training that paired the novel cue with ostensive signals, infants continued orienting with these cues and learning about the cued multimodal events, even though the face was no longer present. Critically, this learning effect was not due to the mere exposure length of the novel cue. Without the initial pairing of the ostensive signal with the novel cue, infants displayed no specific multimodal learning from this cue. In both the *Social Non-Ostensive Signaling* and *No Signaling* experiments, infants demonstrated no specific multimodal learning (only general spatial learning of either the cued locations or of the objects) and no transfer of learning to the new multimodal pairs during generalization.

Learning about a novel stimulus by pairing it with a familiar stimulus has been shown to be effective in other studies with human adults and rats (Honey and Hall, 1989; Liljeholm and Balleine, 2010). These studies show that repeated consistent pairing of stimuli lead to *acquired equivalence*, where the properties of one stimulus are generalized to its complement. In the early learning environment, ostensive signals (e.g., eye contact or addressing the child) often precede social attention cues (especially eye gaze and pointing). Given previous work showing that infants interpret these ostensive signals as communicative (i.e., communicating about an upcoming interesting event to learn; Parise et al., 2007; Csibra and Gergely, 2009), as in previous studies showing acquired equivalence, perhaps infants could transfer their prior knowledge about the function of ostensive signals to novel attention cues.

One issue not explored in this study is the possibility of a spectrum of optimal cueing and *learning to learn* from cues during infancy. The results from the *Social Non-Ostensive Signaling* condition seem to fit between the *Ostensive Signaling* and *No Signaling* conditions (very specific and no multimodal learning, respectively), supporting this notion of a possible spectrum. The effectiveness of learning signals and attention cues may be a function of the infant's experience with them. Allowing for increased familiarity with the function of a signal or cue (over hours, days, perhaps even months) may allow infants to use them to learn and be less distracted by them while learning about other objects. Perhaps the current paradigm could calibrate the “optimality” of an attention cue for infant learning, as well as provide information about why individual infants may not learn as well from different cues (see Yurovsky et al., 2012 for a computational model exploring individual differences in cue and object learning based on data from Wu and Kirkham, 2010). While our sample sizes for the three experiments were relatively small (but the effect sizes for most of the critical comparisons were medium to large), an increase in the sample sizes in future work could address the possibility of cue optimality, individual differences, and the few marginal effects we found in this study.

In addition, future studies will have to determine the exact contextual requirements for how ostensive signals support cued learning (e.g., which of the many signals that were conveyed in the face stimulus are necessary). Multiple factors such as mutual gaze and infant-directed speech were absent in the *Social Non-Ostensive Signaling* condition, and present in the *Ostensive Signaling* condition. However, there were many other differences between the two central signals, such as that they were videos of muppets vs. a real person, had male vs. female voices, and had multiple agents vs. one agent. Comparing the *Non-Ostensive* and *Ostensive* conditions indicates that learning from novel cues requires more than a generally social non-ostensive stimulus presented centrally prior to the learning event. However, narrowing down the exact factors by “breaking down” the ostensive signal will be required to draw further conclusions. Different features of the ostensive signal could be also be compared on the basis of infants' overall attention to the central stimulus, where variations in looking time may account for differences in learning about the target objects. Given previous work showing a difference between looking time during training and quality of learning (e.g., Wu and Kirkham, 2010), it is likely that the type of attention paid to the central signal matters more than the amount of attention. While it is important to know which features of an ostensive signal boost learning, such experiments are beyond the scope of this paper, which rather focused on demonstrating the effect that ostensive signals have on learning the function of a new attention-orienting cue.

While it is unclear *which aspects of* the ostensive signal promoted better multimodal learning, we clearly demonstrate that the presence of ostension can facilitate learning from a new attention-orienting cue. Humans use a variety of cues to communicate what should be learned in the environment; some, such as gaze cues, are readily used by infants months after birth, while others, such as pointing or arrows, take longer to learn about. Our results provide an explanation for *how* infants could learn to learn from novel attention cues—by first pairing them with ostensive signals. We believe the ostensive signals helped infants to discover the function of the novel cue and learn about the cued objects. We demonstrated that this newly acquired function generalized beyond the initial training conditions in a similar way that pointing and arrows eventually are used even when not accompanied by ostensive signals. In this way, ostensive signals may help infants eventually learn to use other attention cues they did not understand earlier in life. The present findings provide an important first step toward elucidating an emerging ability of learning to learn from cues, which extends beyond the documentation of *which* cues guide attention and learning during infancy to propose a mechanism for *how* this learning occurs.

## Author contributions

Conceived and designed the experiments: Rachel Wu, Teodora Gliga, Natasha Z. Kirkham. Performed the experiments: Rachel Wu, Kristen S. Tummeltshammer. Analyzed the data: Rachel Wu. Wrote the paper: Rachel Wu, Teodora Gliga, Kristen S. Tummeltshammer, Natasha Z. Kirkham.

### Conflict of interest statement

The authors declare that the research was conducted in the absence of any commercial or financial relationships that could be construed as a potential conflict of interest.
